# Quantification of Chest Wall Asymmetry in Healthy Females Using Standardized Computed Tomography–Derived Curve Modeling: A Proof of Concept

**DOI:** 10.1093/asjof/ojaf116

**Published:** 2025-09-23

**Authors:** Erin N Abbott, Anvith P Reddy, Annika Coleman, Madeline V Brandt, Kim L Sandler, Patrick G Maxwell, Galen Perdikis, Allen Gabriel

## Abstract

**Background:**

Anterior chest asymmetry is common, with potential implications for aesthetic and reconstructive breast surgery. However, current assessments rely on visual inspection or linear measurements, and few studies offer a reproducible, quantitative framework for analyzing skeletal asymmetry in healthy populations.

**Objectives:**

The aim of the study is to develop and evaluate a proof-of-concept methodology for quantifying anterior thoracic skeletal asymmetry using standardized, computed tomography (CT)-derived curve segmentation and polynomial modeling.

**Methods:**

Chest CT scans from 50 female patients aged 18 to 45 with a BMI of 20 to 25 were evaluated using semi-automated segmentation. The right and left chest wall curvatures were extracted in both a horizontal and vertical plane at the level of the fourth rib insertion and one-fourth of the maximum thorax width, respectively.

**Results:**

The left chest wall displayed significantly greater outward projection than the right chest in both planes (*P* < .001). The most pronounced differences were observed from 2 to 5 cm from the sternum in the horizontal plane and 0 to 1 cm from the manubrium in the vertical plane. Individual assessments showed that >65% of patients exhibited leftward prominence in the horizontal and vertical planes. The estimated mean volume discrepancy between the left and right chest walls was 19.3 cc.

**Conclusions:**

This study introduces a reproducible, curve-based methodology for quantifying skeletal chest wall asymmetry using CT images. In this healthy female cohort, a statistically significant leftward asymmetry was common. Although the volumetric difference may be small and not clinically significant in isolation, these findings support individualized skeletal assessment as a basis for surgical planning, warranting future studies with 3D modeling and more diverse populations.

**Level of Evidence: 4 (Therapeutic):**

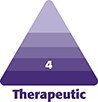

Breast asymmetry is a well-established concern in both aesthetic and reconstructive breast surgery, as it may influence surgical planning and patient satisfaction. Existing literature highlights the psychological impact of asymmetry, including decreased self-esteem, altered body image, and the financial burden associated with additional revision procedures.^1-3^

Anterior chest wall asymmetry, defined as variation in the contour, projection, or alignment of the ribcage or sternum, results from various etiologies. Outside of normal anatomic variation within the population, these include both congenital anomalies and acquired deformities. Studies across plastic surgery, thoracic surgery, and radiology have used computed tomography (CT) scans and 3D surface imaging to assess asymmetry, with an estimated prevalence ranging from 9% to 77%. Collectively, these studies suggest that asymmetry is both prevalent and quantifiable with modern imaging techniques.^4-6^ Despite this, a standardized quantitative approach to skeletal chest wall asymmetry remains lacking, with many studies relying on subjective visual assessments or imprecise linear measurements. Moreover, clinical evaluation and management of breast asymmetry have traditionally focused on soft tissue discrepancies, often overlooking any underlying skeletal contributions.

To contribute to this emerging body of work, we conducted a proof-of-concept study specifically focused on skeletal variation to determine whether mathematically significant curvature and volumetric differences could be identified using standardized measurements with mathematical fitting. We aim to quantify inherent skeletal asymmetry between the left and right chest walls in both the axial and sagittal planes. These findings provide a methodological foundation for future surface-based modeling and more sophisticated volumetric analyses.

## METHODS

### Selection and Screening

This study was approved by the Vanderbilt IRB number #230893. Research Derivatives, a database including all patients at the institution from November 2017 to present, was queried in October 2024 for female patients with chest CT scans (CPT codes: 71250, 71260, 71270, 71271, and 71275), including both contrast-enhanced and non-contrast studies of the chest or chest–abdomen–pelvis. Scans were performed for various clinical indications unrelated to chest wall assessment, and asymmetry was evaluated retrospectively. Patients aged 18 to 45 with a BMI of 20 to 25 at the time of the scan were randomly selected for screening. Exclusion criteria included a prior diagnosis of scoliosis, pectus carinatum, pectus excavatum, or other congenital deformities, and pregnancy at the time of the scan. Additional exclusions included a history of chest wall, vertebral, or rib trauma; pneumothorax; chronic pulmonary conditions such as cystic fibrosis or chronic obstructive pulmonary disease; and a history of breast cancer, breast radiation, reconstruction, or implants. Fifty patient scans were included in the final analysis.

### Image Segmentation

**Figure 1.**
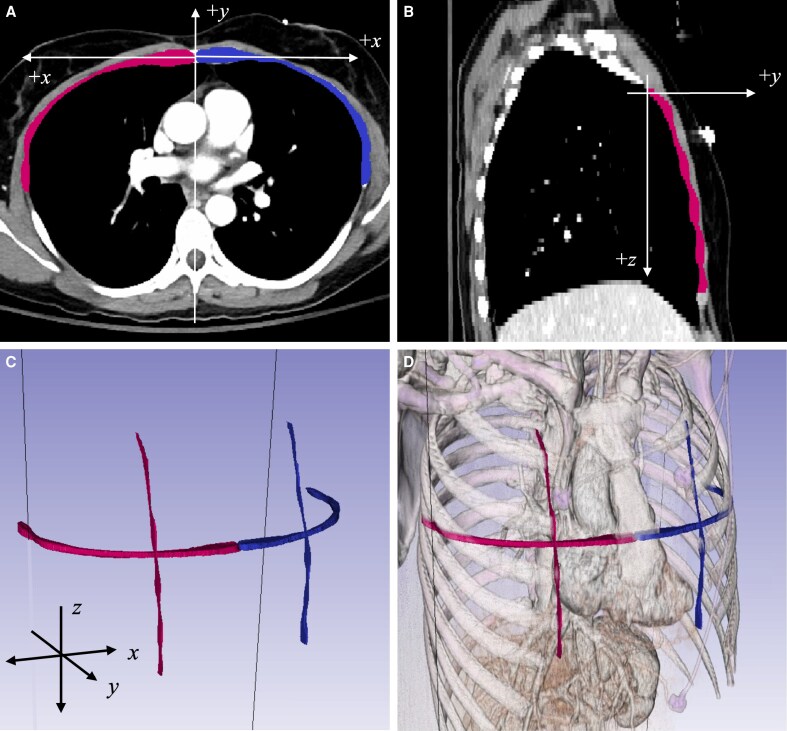
Segmentation of the bony thorax. (A) Axial CT slice showing the horizontal segmentation plane at the level of the fourth rib insertion into the sternum. The thorax is divided into right and left segments. (B) Sagittal CT slice demonstrating the vertical segmentation plane, placed at one-quarter of the thorax width at the same level. (C) Three-dimensional representation of the segmentation planes with orientation shown in *x*, *y*, and *z* axes. (D) Anatomical 3D rendering of the segmentation planes overlaid on thoracic anatomy, excluding chest wall musculature.

Image processing was conducted on 3 mm axial CT scans using the Simpleware segmentation platform (Version 2024.06; Synopsys, Inc., Sunnyvale, CA). Semi-automated methods were applied to capture the curvature of the right and left chest walls in both horizontal and vertical planes. All segmentations and measurements were performed in the soft tissue window. Thoracic width was measured on the axial CT scan slice at the level of the fourth rib insertion into the sternum. For the horizontal segmentation, the plane was set at the fourth rib insertion at the sternum (Figure 1A). For vertical segmentation, the segmentation plane was fixed at one-quarter of the thorax width at the level of the fourth rib insertion (Figure 1B). Chest musculature and soft tissue were not included in the segmentation. The segmentation was verified with a 3D anatomic visualization (Figure 1C, D). Centerlines for the left and right horizontal and vertical curves were created and exported as 3D (*x*, *y*, *z*) coordinates. The coordinates were transformed into a standardized Cartesian plane with a static *z* plane for the horizontal curve and a static *x* plane for the vertical curve. The horizontal curve's origin was set at the intersection of the anterior median line and the level of the fourth rib insertion, while the vertical curve's origin was at one-quarter of the thorax width and the superior edge of the manubrium. Following the transformation, the *y*-axis values were extracted at standardized 1-cm intervals from the origin for statistical analysis. The *x*-axis values for the horizontal curves spanned 0 to 10 cm from the origin, and the *z*-axis values for the vertical curves spanned 0 to 12 cm from the origin.

### Statistical Analysis

Using previously published methodology by Hristova et al,^7^ statistical differences between the mean right and left chest wall curves were evaluated. A *χ*^2^ test was applied at standardized intervals to assess pointwise differences in mean curve values.^6^ A significance level of .05 was used for all statistical analyses.

### Curve Fitting for Area and Volume Calculations

Mathematica (Version 14.1; Wolfram Research, Champaign, IL) was used to apply a least-squares polynomial fit to the standardized curvature data. For each 1-cm interval along the *x*-axis (horizontal plane) and *y*-axis (vertical plane), the mean projection values across all patients for each plane were calculated for the left and right chest walls. Polynomial functions were then fitted to these mean curves to model overall chest wall shape and estimate area and volume differences. The degree of the polynomial used for the fit, which is a measure of how well a function fits a data set given the complexity of the function, was chosen to minimize variance. In this case, the maximum degree was set to 5 to limit noise in the model. To calculate the area difference, the definite integral of the difference between the left fit polynomial and the right fit polynomial was taken to calculate the area enclosed by the functions. The area between the mean curves for horizontal and vertical was calculated.

Additionally, the area was calculated between the curves for each of the 50 individual patients. Distribution statistics were performed using the D′Agostino–Pearson *K*^2^ test on the distribution of areas with a positive area indicating more anterior projection on the left, with significance set at a *P*-value of .05. To approximate volumetric differences, each area was multiplied by the orthogonal chest dimension: the mean horizontal curve area was multiplied by the vertical chest height (120 mm), and the mean vertical curve area was multiplied by the horizontal chest width (100 mm). These 2 volumes were averaged to calculate the estimated volume difference between the left and right chest walls. Final volumes were converted from cubic millimeters (mm^3^) to cubic centimeters (cc) using a 1:1000 ratio (1 cc = 1000 mm^3^) to improve clinical interpretability.

## RESULTS

### Summary Statistics

Fifty patients were included in the analysis, with a mean age of 29.6 years (range, 18.6-45.0) and a mean BMI of 22.7 (range, 20.0-24.9). The mean chest wall width was 23.4 cm (range, 18.8-26.5). The mean distance from the sternal midpoint to the vertical curve was 5.9 cm (range, 4.6-7.3) on the left and 5.7 cm (range, 3.8-6.7) on the right, with no significant difference observed (*P* = .13).

### Difference in Mean Chest Wall Curvature

The horizontal chest wall curves differed significantly between the left and right sides throughout their trajectories (*P* < .001). The mean *y*-values were significantly different from 2 to 5 cm from the sternum, with the left chest curve remaining anteriorly projected over a longer distance. The difference of the left vs right mean curves was +1.55 mm at 2 cm from the origin (*P* < .01), +1.94 mm at 3 cm (*P* < .01), +1.84 mm at 4 cm (*P* = .02), and +1.98 mm at 5 cm with no significant differences found at 1 cm or between 6 and 10 cm (Table 1).

**Table 1.** Values and Means at Standardized Points Along Mean Horizontal Curves

Horizontal chest wall slope										
*x* (mm)^a^	*y*, Left (mm)^b^		*y*, Right (mm)		Statistics					
	Mean	SD	Mean	SD	Difference (L vs R)^c^	SE diff	*t*-Value	df	*P*-value	*χ* ^2^
0	0.00	0.00	0.00	1.24	0.00	0.00				
10	0.34	1.09	−0.13	2.08	+0.47	0.23	2.00	98	.05	3.91
20	0.96	1.77	−0.59	3.04	+1.55	0.39	4.01	98	<.01	14.80
30	0.95	2.70	−0.99	4.16	+1.94	0.58	3.38	98	<.01	10.76
40	0.57	3.40	−1.27	5.09	+1.84	0.76	2.42	98	.02	5.65
50	−0.52	4.17	−2.50	6.50	+1.98	0.93	2.13	98	.04	4.41
60	−2.92	4.87	−4.30	7.69	+1.38	1.15	1.20	98	.23	1.43
70	−6.28	6.04	−7.96	8.96	+1.68	1.38	1.21	98	.23	1.45
80	−11.30	7.46	−12.66	10.52	+1.36	1.65	0.82	98	.41	0.67
90	−17.97	9.53	−19.40	13.58	+1.43	2.01	0.71	98	.48	0.51
100	−28.09	13.33	−29.30	1.24	+1.21	2.69	0.45	98	.65	0.20
								Sum of *χ*^2^		43.79
								df		9
								*P*-value		<0.0001

^a^ *x* (mm), distance from sternal origin in millimeters. ^b^*y* (mm), anterior projection in the horizontal plane in millimeters. ^c^Positive values indicate greater projection on the left; negative values indicate greater projection on the right. L, left; R, right; SD, standard deviation; SE, standard error; df, degrees of freedom; *χ*^2^, *χ*^2^ statistic.

Similarly, the vertical chest wall curves showed significant differences between the left and right sides (*P* < .001). Mean *y*-values differed significantly from 0 to 1 cm below the superior border of the level of the superior manubrium, with the left vertical curve demonstrating greater anterior projection than the right. The difference of the left vs right curves at the most superior point of the vertical curve was +2.59 mm (*P* < .001) and +2.05 mm at 1 cm (*P* = .03). The vertical curves were not significantly different over the rest of the course, indicating no significant difference in projection (Table 2).

**Table 2.** Values and Means at Standardized Points Along Mean Vertical Curves

Vertical chest wall slope										
*z* (mm)^a^	*y*, Left (mm) ^b^		*y*, Right (mm)		Statistics					
	Mean	SD	Mean	SD	Difference (L vs R)^c^	SE Diff	*t*-Value	df	*P*-value	*χ* ^2^
0	2.59	4.82	0.00	0.00	+2.59	0.68	3.81	98	<.01	13.45
10	11.19	5.08	9.14	4.22	+2.05	0.93	2.20	98	.03	4.68
20	16.86	5.87	14.80	5.58	+2.06	1.15	1.79	98	.08	3.15
30	21.73	6.97	19.61	6.71	+2.12	1.37	1.55	98	.13	2.36
40	26.45	7.81	24.40	7.56	+2.05	1.54	1.33	98	.19	1.75
50	30.57	8.30	28.27	8.24	+2.30	1.65	1.39	98	.16	1.91
60	33.66	8.96	31.24	9.34	+2.42	1.83	1.32	98	.19	1.72
70	36.33	9.62	34.21	10.31	+2.12	1.99	1.06	98	.29	1.12
80	38.30	10.37	36.54	11.24	+1.76	2.16	0.81	98	.42	0.66
90	39.88	11.12	38.60	12.05	+1.28	2.32	0.55	98	.58	0.30
100	41.08	11.76	39.88	12.74	+1.21	2.45	0.49	98	.62	0.24
110	41.88	12.29	41.13	13.32	+0.75	2.56	0.29	98	.77	0.09
120	42.14	13.45	41.90	14.23	+0.24	2.77	0.09	98	.93	0.01
								Sum of *χ*^2^		31.43
								df		11
								*P*-value		<0.001

^a^ *z* (mm), distance from sternal origin in millimeters. ^b^*y* (mm), anterior projection in the horizontal plane in millimeters. ^c^Positive values indicate greater projection on the left; negative values indicate greater projection on the right. L, left; R, right; SD, standard deviation; SE, standard error; df, degrees of freedom; *χ*^2^, *χ*^2^ statistic.

### Difference in Mean Curvature Area

The best-fit polynomial functions modeling the mean curvature of the left and right chest walls in both horizontal and vertical planes are presented in Table 3.

**Table 3.** Best-Fit Polynomial Functions for the Mean Left and Right Curve

Horizontal polynomials	Left	$-0.00364x+0.00532x^{2}-0.000180x^{3}+1.812\times 10^{-6}x^{4}-8.169\times 10^{-9}x^{5}$
	Right	$-0.0368x+0.000887x^{2}-0.0000119x^{3}-2.2585\times 10^{-7}x^{4}$
Vertical polynomials	Left	$-1.265y-0.0275y^{2}-0.000429y^{3}-3.422\times 10^{-6}y^{4}-1.018\times 10^{-8}y^{5}$
	Right	$-1.0195y-0.0178y^{2}-0.0002562y^{3}-1.983\times 10^{-6}y^{4}-5.799\times 10^{-9}y^{5}$

**Figure 2.**
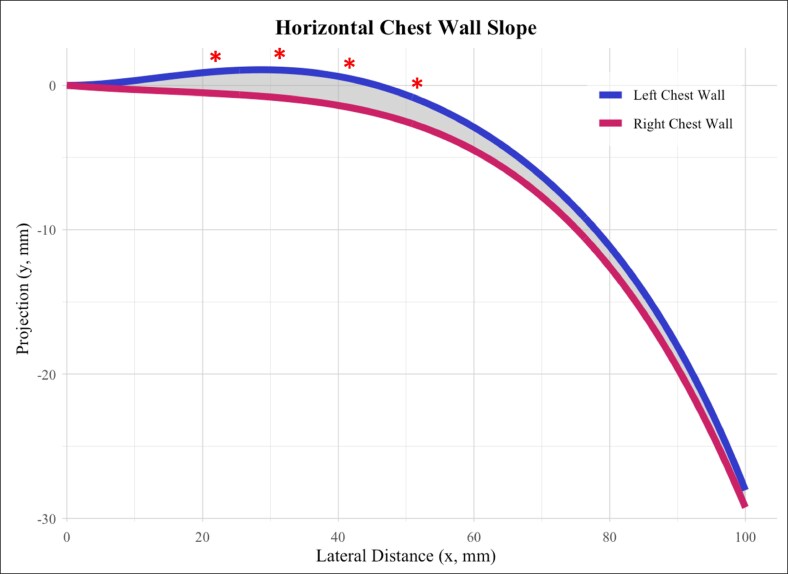
Horizontal best-fit polynomials comparing mean left and right chest wall curvature. Best-fit curves for the left and right chest wall contours are shown, with the shaded area representing the difference between curves in the horizontal plane. Statistically significant differences between standardized 1-cm points are indicated by red stars.

**Figure 3.**
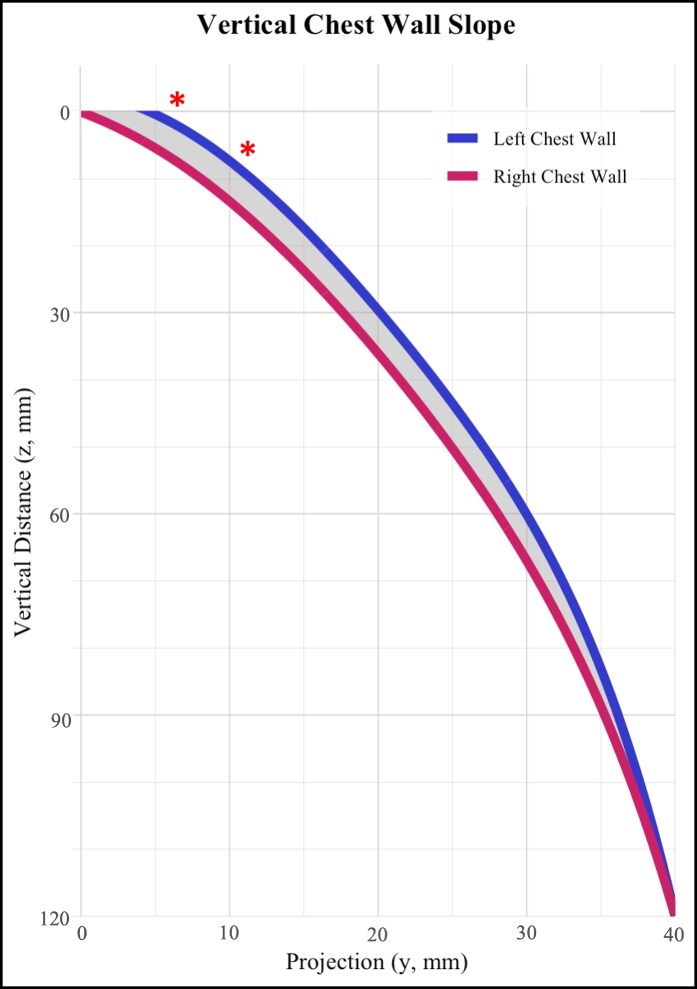
Vertical best-fit polynomials comparing mean left and right chest wall curvature. Best-fit curves for the left and right chest wall contours are shown, with the shaded area representing the difference between curves in the vertical plane. Statistically significant differences between standardized 1-cm points are indicated by red stars.

The area difference between the left and right horizontal best-fit curves was 143 mm^2^ over the 100-mm distance (Figure 2). The difference between the left and right vertical best-fit curves was 202 mm^2^ over the 120-mm distance (Figure 3).

### Difference in Mean Individual Curvature Area

**Figure 4.**
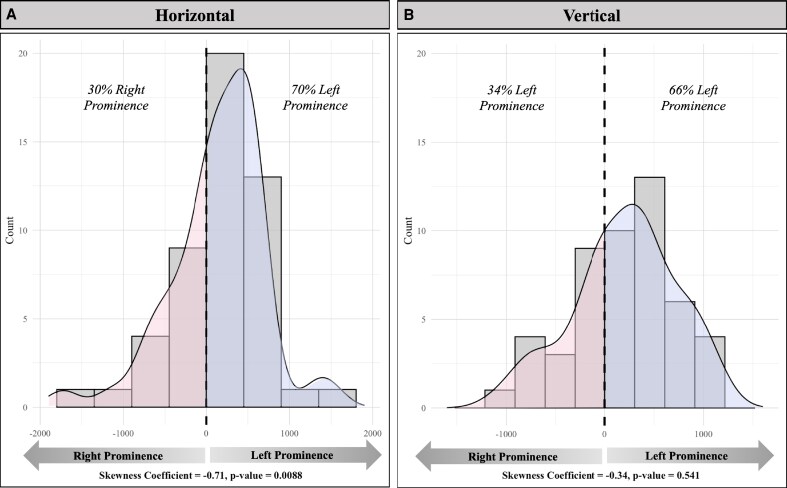
Histograms of individual patient chest wall prominence. (A) Horizontal plane distribution showing the relative anterior projection of left vs right chest walls. (B) Vertical plane distribution showing relative anterior projection of left vs right chest walls. Percentages denote the proportion of patients with left- or right-sided prominence, and skewness coefficients with *P*-values are provided below each histogram.

In addition to fitting polynomial curves to the average chest wall curvature, we examined individual variation within the study population. For the horizontal curves, 35 out of 50 patients (70%) had a positive area difference, indicating greater anterior projection of the left chest wall compared with the right. The remaining 15 patients (30%) showed a negative area difference, reflecting greater anterior projection on the right side. The distribution of area differences was left-skewed, with a skewness coefficient of −0.71 (*P* < .01) (Figure 4A). For the vertical curves, 33 of 50 patients (66%) demonstrated a positive area difference, indicating greater anterior projection on the left side, while 17 patients (34%) showed the opposite pattern. The corresponding skewness coefficient was −0.34 (*P* = .54), indicating a more symmetric distribution (Figure 4B).

### Volume Estimation

Crude volume estimates were derived from the area between the left and right best-fit curves, multiplied by the height of the chest wall in both horizontal and vertical planes. After height multiplication and unit conversion to cc, the estimated volume difference was 14.3 cc in the horizontal plane and 24.2 cc in the vertical plane. Averaging these yielded an overall estimated volume discrepancy of 19.3 cc, with greater projection of the left chest wall. Individual patient volume discrepancies ranged from 62.6 cc of right-sided to 146.3 cc of left-sided prominence.

## DISCUSSION

In a limited, healthy female cohort, we identified statistically significant anterior chest wall asymmetry, with greater projection typically observed on the left side. The greatest differences were observed between 2 and 5 cm from the sternum horizontally and 0 to 1 cm from the superior border of the manubrium along the vertical axis. Although 70% and 65% of individuals showed greater left-sided projection in the horizontal and vertical planes, respectively, the vertical dimension showed more pronounced inter-individual variability. The mean estimated volume difference necessary to balance anterior projection was ∼20 cc, with individual differences ranging from −62.6 to +146.3 cc, reflecting substantial variability in chest wall anatomy.

This study presents a reproducible, curve-based method for evaluating anterior thoracic skeletal asymmetry, using CT-derived contours and polynomial fitting. By isolating skeletal features across orthogonal planes, this method offers detailed spatial resolution that can inform implant selection, flap design, and asymmetry correction. In contrast to broader indices and surface imaging methods, our approach integrates statistical modeling and anatomic segmentation to improve precision in personalized surgical planning.

Chest wall asymmetry is well documented in congenital deformities and skeletal anomalies. In a series of 300 patients with pectus excavatum, 80% showed right-sided sternal depression, with only 10% demonstrating midline positioning.^8^ In scoliosis, particularly adolescent idiopathic scoliosis, anterior chest wall deformity has been shown to affect cosmesis, with studies documenting improved symmetry following surgical correction.^9,10^ Similarly, anterior thoracic hypoplasia (ATH), a subtle and often underdiagnosed skeletal condition, has been noted to contribute to anterior volume deficiencies that may compromise reconstructive outcomes.^11^ These conditions highlight the need for accurate thoracic contour characterization, which current measurement techniques often fail to provide due to limited granularity.

Conventional assessments of chest wall asymmetry often rely on broader indices, such as sternal deviation, rib measurements, and Haller or Asymmetry Index scores. Although effective for identifying severe deformities, these methods assume bilateral symmetry and overlook localized projection differences essential for aesthetic and reconstructive planning.^12,13^ Additionally, minor asymmetries often go unreported in clinical radiology unless linked to trauma, malignancy, or overt deformities, leading to limited awareness of subtle but clinically relevant skeletal variation.^14^

To address these limitations, imaging-based approaches such as CT and MRI with 3D reconstruction have been developed to simulate thoracic morphology and analyze surgical correction.^6,15-17^ Other studies have constructed chest contours from CT or 3D surface imaging to evaluate morphology or establish diagnostic benchmarks.^18-21^ Although 3D surface scanners provide radiation-free imaging, their limited volumetric precision and high cost restrict widespread clinical adoption.^19,20,22^

Even in the absence of overt pathology, chest wall asymmetry is prevalent in healthy individuals and is increasingly recognized as relevant to aesthetic surgical planning. Hirsch and Brody identified measurable volumetric asymmetries in all 50 women assessed by CT, including differences in lateral width, anterior–posterior diameter, and internal angles.^23^They concluded that chest wall slope may influence postoperative breast projection and cleavage contour. Kolasinski et al similarly reported projection asymmetry in 84% of women, noting its relevance to implant selection and surgical planning.^24^ Gabriel et al further demonstrated, using 4D photographic analysis, that all women in their study exhibited some degree of musculoskeletal or soft tissue asymmetry. Based on these findings, they advocated for individualized preoperative assessments.^25^ Beyond the presence of asymmetry, several studies report a consistent pattern of left-sided thoracic prominence. Henseler et al reported that the left nipple-to-midline distance was significantly greater than the right, while Losken et al found left-sided dominance more frequent in patients with higher BMI and larger chest circumference, supporting our decision to use a BMI-matched cohort.^26,27^ Hafezi et al found the left chest wall to be wider, correlating with breast soft tissue volume.^28^ Together, these studies highlight the frequency and variability of asymmetry even in healthy populations.

The increasing awareness of asymmetry in healthy individuals has prompted calls for systematic, individualized preoperative evaluation in aesthetic surgery. Attention to thoracic contour is increasingly emphasized in preoperative planning, as it can inform implant selection and improve surgical symmetry outcomes.^24,25,29^ Rinaldi specifically notes that understanding thoracic morphology is critical for achieving natural implant results, particularly in cases of subtle skeletal conditions like ATH, which often go undiagnosed but affect surgical outcomes.^30^ Our findings reinforce calls for anterior chest wall evaluation.

Although the estimated mean volume discrepancy of approximately 20 cc was observed, it represents a clinically relevant portion of standard implant volume increments, which typically vary in steps of 20 to 30 cc. Even subtle asymmetries in skeletal projection can influence implant selection or flap volume on each side, as well as the overall perception of symmetry, particularly in lean patients or those undergoing revision surgery. While this level of assessment may not be necessary for every patient, its utility may be greatest in individuals with preoperative dissatisfaction, visible thoracic asymmetry, or a history of reconstruction. In this context, skeletal contour analysis may be most impactful when applied selectively as a targeted preoperative tool in patients where asymmetry is either suspected or visually apparent. Multiple studies have emphasized the need for systematic preoperative evaluation of the thorax to better inform patients and establish realistic expectations.^24,25,29,30^ These findings highlight the potential role of scalable quantitative asymmetry analysis for expectation setting and surgical decision-making. Importantly, these volume discrepancies should not be used as universal thresholds but rather as a reminder of the need for patient-specific assessment. Ultimately, this methodology provides a scalable foundation for incorporating skeletal assessment into personalized surgical planning, especially as imaging and modeling technologies evolve.

Our study is primarily limited by the modest sample size, which reflects the resource-intensive nature of semi-automated segmentation and the intent to establish proof-of-concept within a controlled cohort. The narrow inclusion criteria were intentionally selected to establish a controlled, healthy baseline cohort, improving internal validity but limiting generalizability. Inclusion of higher-BMI individuals and male patients will be necessary to broaden applicability beyond the typical aesthetic breast surgery population. Second, our volume estimates were derived from planar integration rather than true 3D modeling and should therefore be considered approximate. Third, although our analysis was conducted on previously obtained CT scans, the radiation burden associated with CT limits its practicality for elective asymmetry evaluation in healthy patients. Refinement of this method for non-ionizing imaging platforms may enhance its clinical applicability. Finally, the observed left-sided prominence may reflect physiologic variation or subclinical skeletal anomalies such as ATH. In addition, leftward prominence may be influenced by underlying anatomic factors such as the position of the heart. These possibilities should be explored with larger cohorts, advanced surface rendering, and broader validation across diverse populations to better determine the clinical significance of skeletal asymmetry and its impact on aesthetic and reconstructive outcomes.

## CONCLUSIONS

This proof-of-concept study presents a reproducible method for quantifying anterior chest wall asymmetry using CT-derived curve segmentation combined with polynomial fitting. In this narrow cohort of healthy females with BMI 20 to 25, we observed a consistent trend toward greater anterior projection of the left chest wall in both horizontal and vertical planes. This method provides a scalable framework that may support decision-making in cases of skeletal asymmetry, particularly regarding implant selection and preoperative counseling.
